# Supplementary material for: Investigating the sources of variable impact of pathogenic variants in monogenic metabolic conditions
Source: Nat Commun. 2025 Jun 5;16:5223. doi: 10.1038/s41467-025-60339-7 (PMC12141715; doi:10.1038/s41467-025-60339-7)
Supplement: Supplementary file 1 — Supplementary Information [file 41467_2025_60339_MOESM1_ESM.pdf]

Supplemental Methods

Table 1: Pathogenic variant curation methods

**Pathogenic variant group**	**Identification method**
Curated pathogenic variants	Variants identified by Goodrich, et al., and Mirshahi, et al., using ACMG/AMP criteria and blinded testing by reviewers
ClinVar-weak	Variants in ClinVar with at least one report of “pathogenic” or “likely pathogenic”, but may contain additional conflicting reports
ClinVar-strong	Variants in ClinVar with only reports of “pathogenic” or “likely pathogenic”

Testing for epistasis via PRS

Testing for genetic epistasis occurring between common background genetic variation and monogenic variant carrier status was completed using the model, $y=\Sigma G\cdot\beta_G+C\cdot\beta_C+\Sigma G\cdot C\cdot\beta_{CxG}+\epsilon$, where y is the phenotype of interest, G represents common genetic variation and its associated effect β_G on the phenotype of interest, C is an indicator if an individual is carrying a pathogenic variant and β_C is the effect size of the monogenic variant on the phenotype of interest, and G·C is the interaction between common background variation and carrier status (i.e., genetic epistasis) with its associated effect size on the phenotype of interest, β_{CxG}. Covariates, such as age, sex, and the individual’s first 10 genetic PCs are also adjusted for in this model. One method this project employed to test for genetic epistasis was to use PRS as a proxy for $\Sigma G\cdot\beta_G$, leading to the model $y=\beta_{PRS}\cdot PRS+C\cdot\beta_C+C\cdot PRS\cdot\beta_{CxPRS}+\epsilon$. Age, sex, and the first 10 genetic PCs were adjusted for in this model.

Additional FAME information

We have defined the target gene(s) proximal region as the specific physical genome region that encompasses the genes we are interested in when analyzing pathogenic variants. By employing this definition, we have divided the $N\times M$ array genotype matrix ($\boldsymbol{G}$) into two separate matrices: the $N\times M_1$ gene-proximal SNP matrix ($\boldsymbol{G_1}$) containing all the SNPs within the physical coverage of the target genes in array SNP data, of which the pathogenic variants are of interest. For example, the target genes of interest can be *LDLR*, *PCSK9*, *APOA5*, and so on. The remaining $N\times M_2$ gene-distal SNP matrix is $\boldsymbol{G_2}$. (We have $M = M_1 + M_2$). As a result, our model incorporates the additive effect of both the $\boldsymbol{G_1}$ and $\boldsymbol{G_2}$ as well as the interactive effect between the pseudo gene of interest ($\boldsymbol{C_t}$) and the $\boldsymbol{G_2}$. The model assumption is formally defined as:

$$\begin{aligned}\boldsymbol{y} &= \sum_{b=1}^{2}\boldsymbol{G}_b\boldsymbol{\beta}_b+\boldsymbol{E}_t\boldsymbol{\alpha}_t+\boldsymbol{\epsilon}\\ \boldsymbol{\epsilon} &\sim \mathcal{N}(\boldsymbol{0},\sigma_e^2\boldsymbol{I}_N)\\ \boldsymbol{\beta}_b &\sim \mathcal{N}(\boldsymbol{0},\frac{\sigma_{G_b}^2}{M_b}\boldsymbol{I}_{M_b}), b\in\{1,2\}\\ \boldsymbol{\alpha}_t &\sim \mathcal{N}(\boldsymbol{0},\frac{\sigma_{C\times G,t}^2}{M_2}\boldsymbol{I}_{M_2})\end{aligned}$$

Here $\mathcal{N}(\boldsymbol{\mu},\boldsymbol{\Sigma})$ defines the normal distribution with mean $\boldsymbol{\mu}$ and covariance matrix Σ. $\boldsymbol{E}_t$ denotes an $N\times M_2$ pseudo-gene by genetic interaction matrix defined as $\boldsymbol{E}_t=\boldsymbol{C}_t\odot\boldsymbol{G}_2$ where $\odot$ the row-wise Kronecker product. Here $\boldsymbol{C_t}$ represents the target pseudo gene of interest and denotes whether individuals carry a burden of pathogenic variants at the target gene *t* defined as follows: $\boldsymbol{C_t}$=0 if individual *i* does not carry any relevant pathogenic variants at target genes, and $\boldsymbol{C_t}$=1 if individual *i*

carries at least one pathogenic variant at the target gene(s) *t*, as detected from the Whole Exome Sequencing (WES) dataset. y denotes the residualized phenotypes as an *N*-vector, which was obtained by taking the original phenotype and regressing out the fixed effect of the target pseudo-gene, together with the top 20 PCs, age, and sex. We use the same notation as the previous section, β_C, to denote the fixed effect of the target pseudo-gene. In this model, σ_e^2, $\sigma_{G_1}^2$, $\sigma_{G_2}^2$, and $\sigma_{C\times G,t}^2$ are the residual variance, additive genetic variance at each bin ($\sigma_{G_1}^2 + \sigma_{G_2}^2 = \sigma_G^2$), and the marginal epistasis variance components, respectively. $\beta_b$denotes the additive effects of SNPs that are proximal and distal to the target gene(s), while α_t denotes the interaction effects between target pseudo gene *t* and each of the SNPs in the gene-distal SNP matrix (***G*₂**). Full details can be found in Fu, et al., 2023.[22]

Supplemental Figures

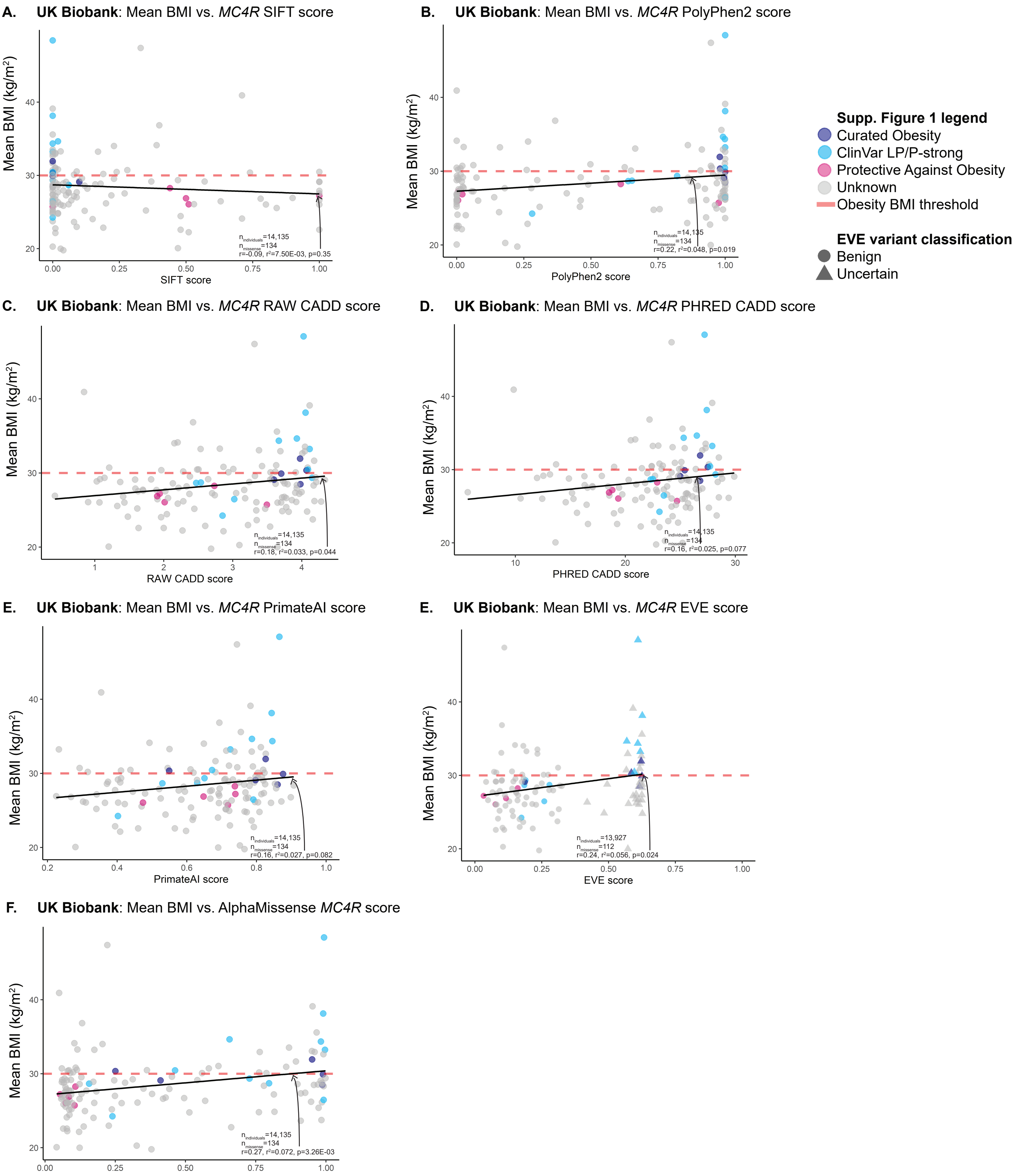

Supplemental Figure 1: Additional variant pathogenicity predictors do not predict mean phenotype as accurately as ESM1b. Phenotype correlations were also compared against additional variant pathogenicity prediction methods (**A**-SIFT, **B**-PolyPhen2, **C**-RAW CADD, **D**-PRED CADD, **E**-PrimateAI, **F**-EVE, **G**-AlphaMissense). These methods have lower Pearson correlations with mean BMI compared to ESM1b and do not differentiate between GOF and LOF missense variants in *MC4R*. EVE also does not have full coverage of all *MC4R* missense variants. p-values generated based on two-sided Pearson correlation tests as described in the Methods.

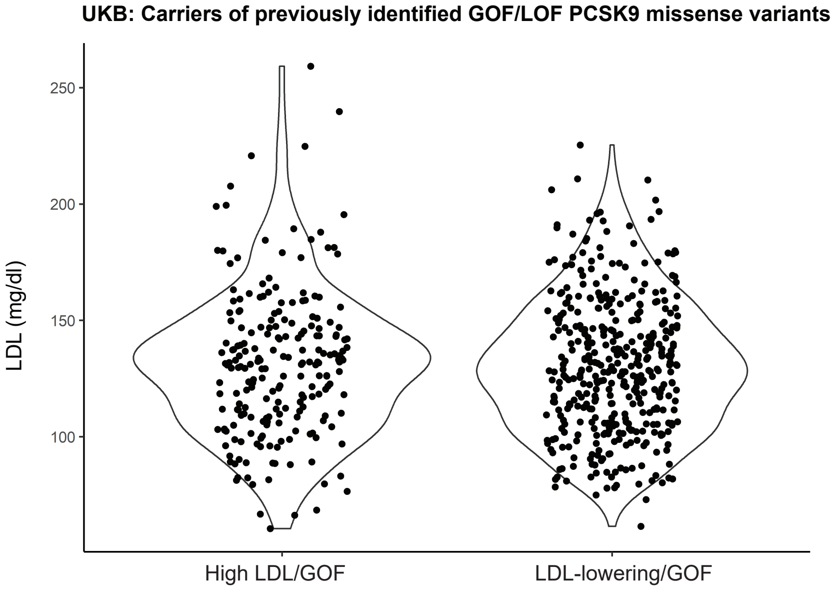

Supplemental Figure 2: Carriers of GOF/LOF *PCSK9* missense variants do not have significantly different LDL levels in UKB. Carriers of *PCSK9* GOF (n=216) and LOF (n=398) missense variants were identified. After adjusting for age, sex, and 1st 10 PCs, carrying a GOF or LOF variant was not significantly associated with LDL levels within these carriers.

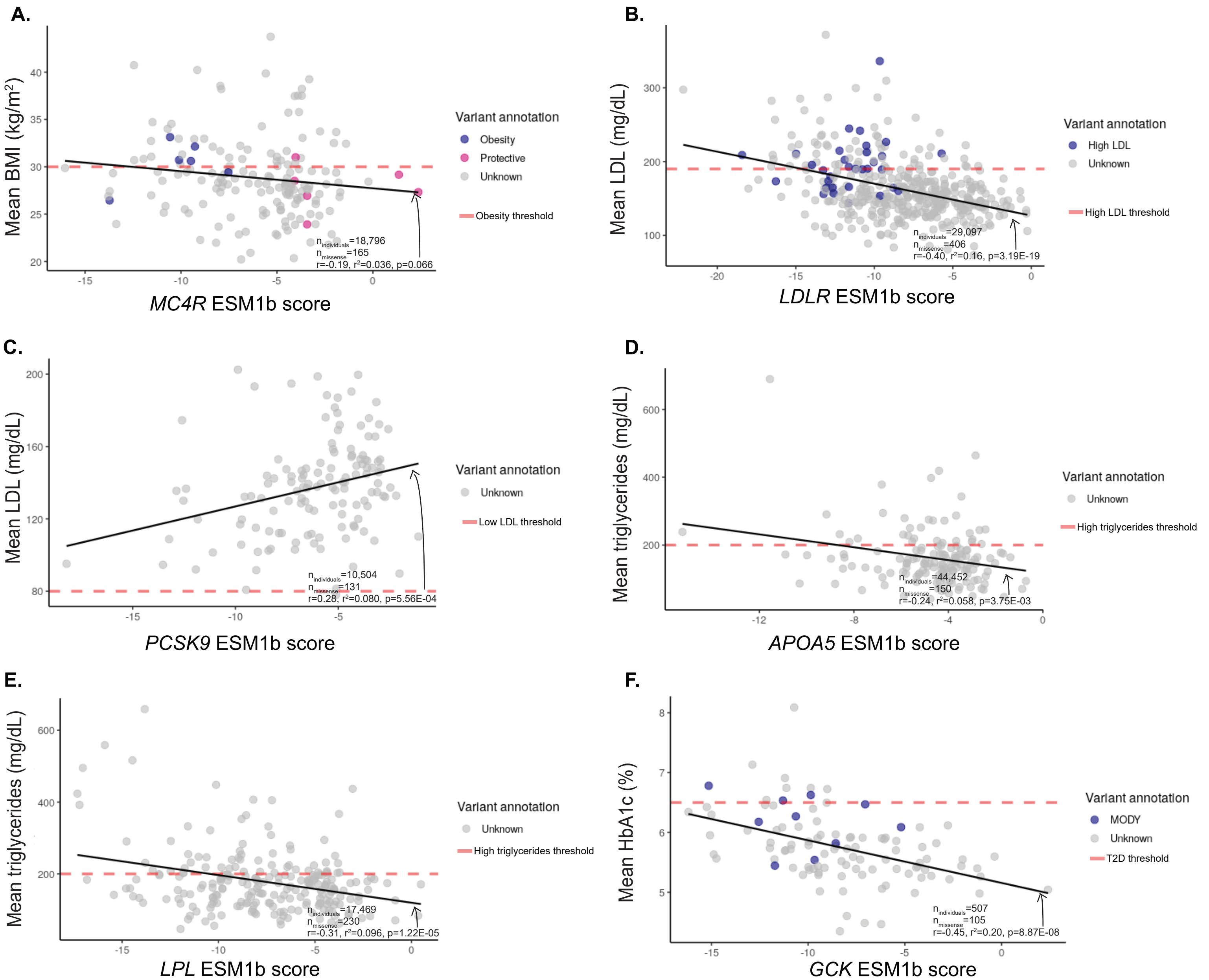

Supplemental Figure 3: Mean phenotype-ESM1b correlations replicate in UKB 500k exomes. 5/6 mean phenotype-ESM1b correlations replicate in the 500k exomes: *LDLR* **(B)**, *PCSK9* **(C)**, *APOA5* **(D)**, *LPL* **(E)**, and *GCK* **(F)**. *MC4R* **(A)** BMI- ESM1b correlation approached significance. Replication completed in individuals only in the UKB 500k exomes release and not in the 200k exomes release. p-values generated as described in Methods.

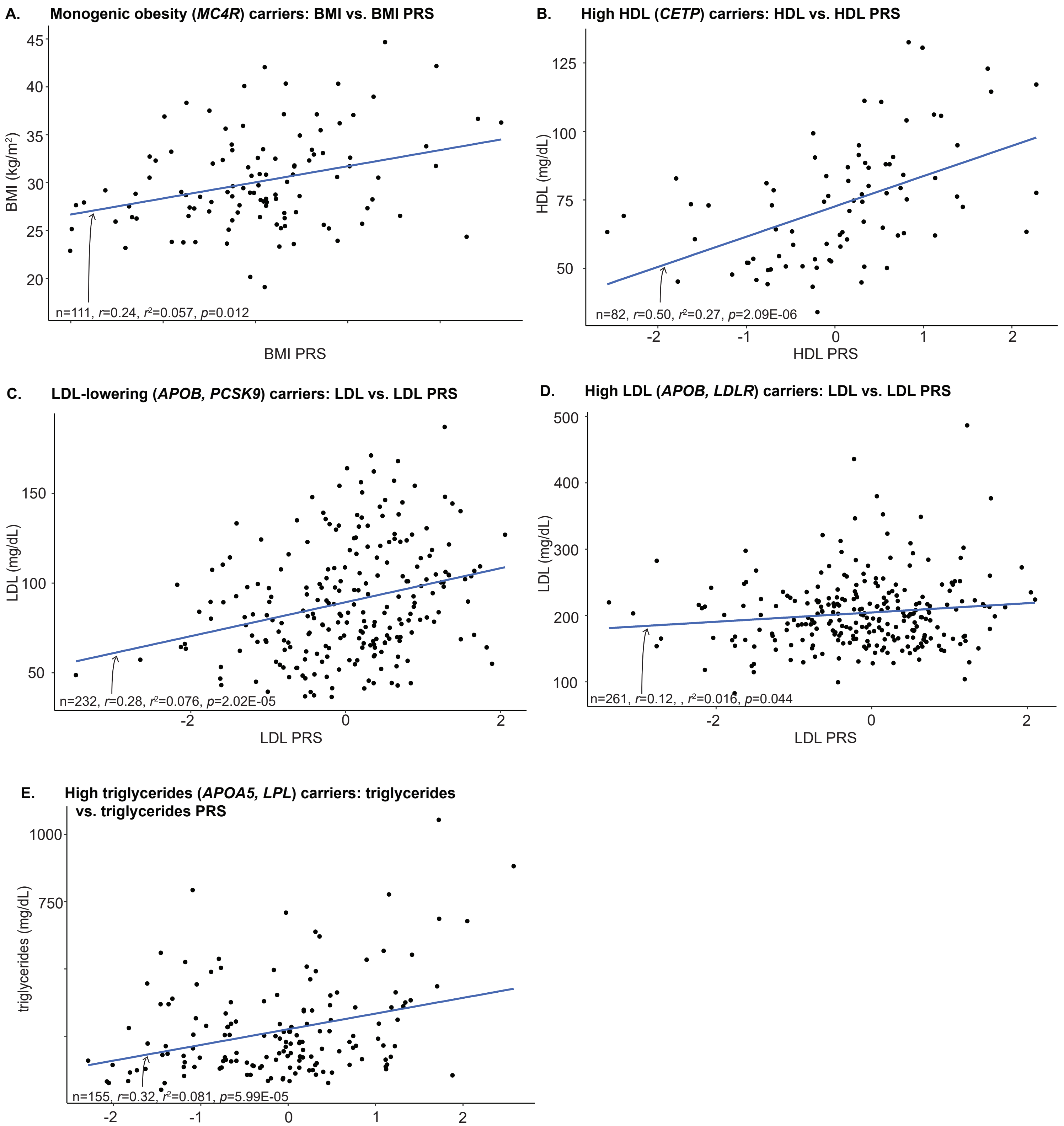

Supplemental Figure 4: PRS contributes to phenotypic variance even within carriers of pathogenic variants. After adjusting for sex, age, first 10 genetic PCs, and Bonferroni corrections ($p<0.05/6=0.0083$) within linear regressions, corresponding carrier PRS for unrelated, European obesity (A; BMI PRS $\beta=1.68$, $p=5.60\text{E-}03$), high HDL (B; HDL PRS $\beta=9.79$, $p=1.57\text{E-}06$), LDL-lowering (C; $\beta=9.87$, $p=3.18\text{E-}06$), and high triglycerides (E; $\beta=62.46$, $p=1.33\text{E-}05$) carriers were significant. High LDL carriers' corresponding LDL PRS approached significance (D; $\beta=6.76$, $p=0.028$). T2D status of MODY carriers was predicted with T2D PRS after adjusting for age, sex, and first 10 genetic PCs; T2D PRS was not significant in this model ($\beta=0.44$, $p=0.15$). Two-sided Pearson correlation test result are shown on each plot.

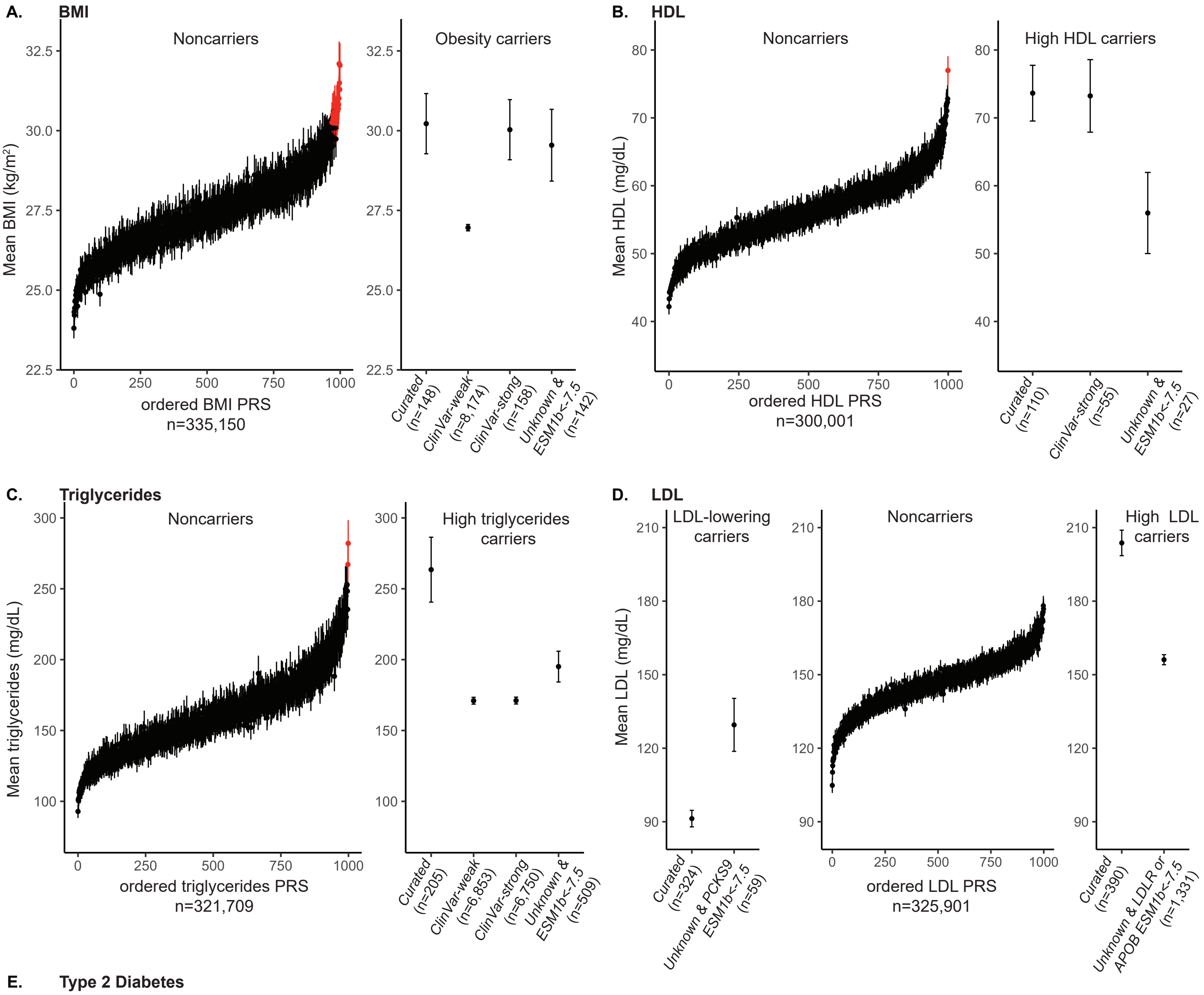

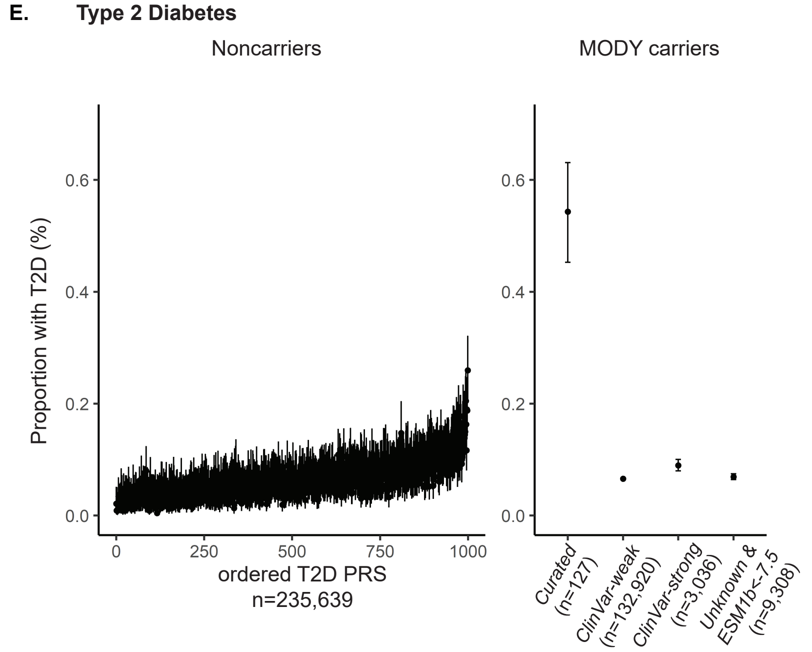

Supplemental Figure 5: Comparison of differential definitions of carrier status and noncarriers by 0.1% bins of PRS. Noncarrier 1000th-tile bins with mean phenotype more severe than carriers highlighted in red: **(A)** monogenic obesity, **(B)** high HDL, and **(C)** high triglycerides. Carriers of curated high LDL and LDL-lowering **(D)** and MODY **(E)** variants have more severe phenotypes than noncarriers in extremes of PRSs. Identification of additional carriers were based on differing levels of stringency for ClinVar filtering ("ClinVar-weak" or "ClinVar-strong") or ESM1b likely pathogenic scoring (ESM1b<-7.5). Red indicates PRS bins where mean phenotype is more severe that curated carriers' mean phenotype.

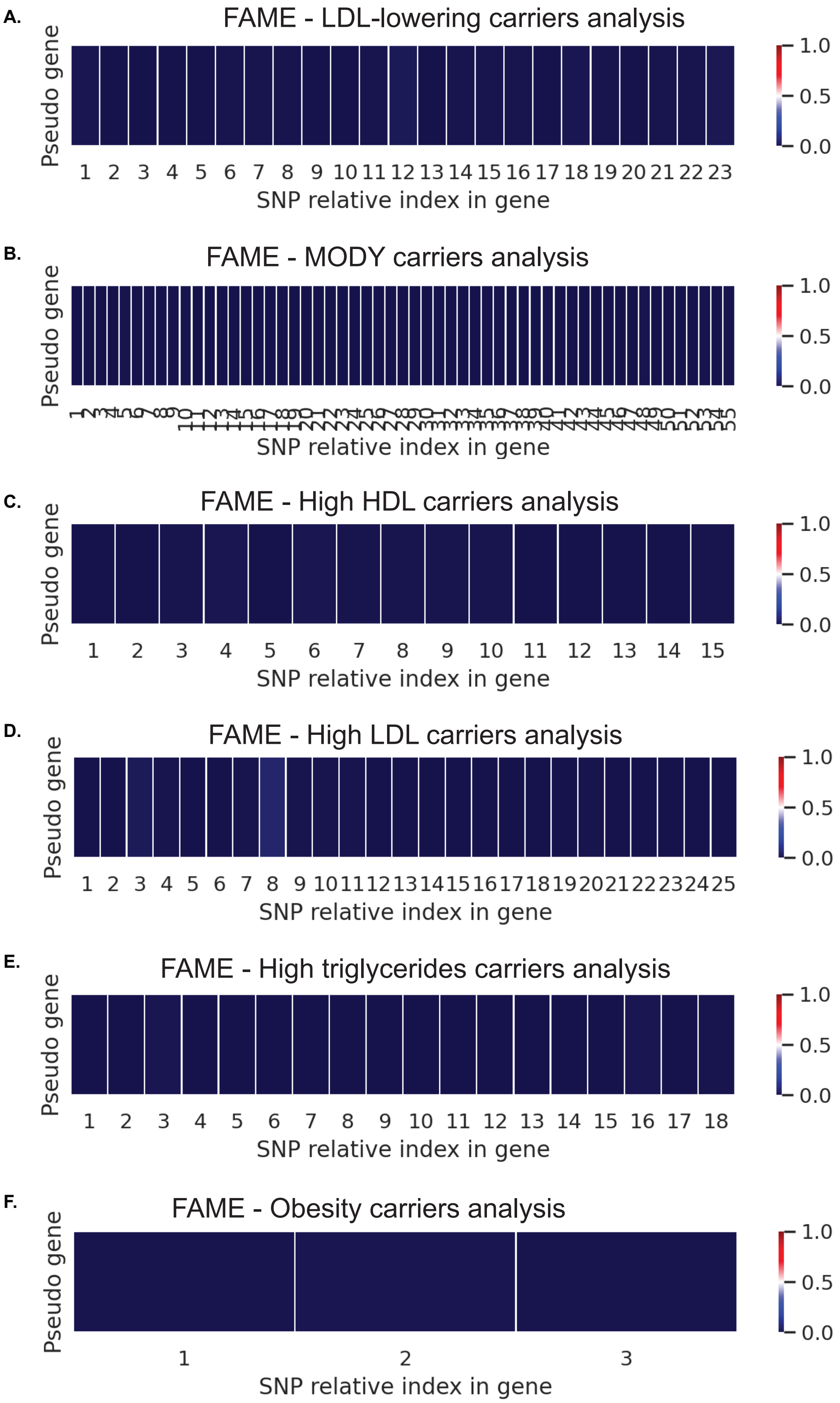

Supplemental Figure 6: FAME marginal epistasis results are unlikely to be unaffected by LD structure. Pearson correlations were calculated between pseudo-genes and single nucleotide polymorphisms (SNPs) in the same region of the curated **(A)** LDL-lowering, **(B)** MODY, **(C)** High HDL, **(D)** High LDL, **(E)** high triglycerides, and **(F)** monogenic obesity carriers. Heat maps shown here represent the absolute value of the correlations.
