# Supplementary material for: Investigating the sources of variable impact of pathogenic variants in monogenic metabolic conditions
Source: Nat Commun. 2025 Jun 5;16:5223. doi: 10.1038/s41467-025-60339-7 (PMC12141715; doi:10.1038/s41467-025-60339-7)
Supplement: Supplementary file 2 — Description of Additional Supplementary Files [file 41467_2025_60339_MOESM2_ESM.pdf]

Description of Additional Supplementary Files

File Name: Supplemental Data 1.
Description: Summary of BioMe biobank participants. Replication of ESM1b results were performed in the BioMe biobank.

File Name: Supplemental Data 2.
Description: Detailed annotations of clinical, monogenic variants used in this study. Genomic position (hg38), functional effect, gnomAD exomes allele frequencies, and number of UKB 200k exomes individuals identified as carriers of the variants are given in this table.

File Name: Supplemental Data 3.
Description: All results from gene variant pathogenicity predictor methods and mean phenotype correlations. Variant pathogenicity methods include ESM1b, SIFT, PolyPhen2, RAW CADD, PHRED CADD, AlphaMissense, and PrimateAI. Correlations were completed on everyone in the UKB 200K exomes ("individuals_included=everyone") or European and unrelated individuals ("individuals_included=european&unrelated") Respective PRS was regressed out for only European and unrelated individuals ("PRS regressed out (T/F)=1)". R^2, standard error (SE), and p-values from two-sided correlation tests given without adjusting for multiple-testing.

File Name: Supplemental Data 4.
Description: Noncarriers in extremes of PRS percentiles show more extreme phenotypes than carriers. Exact 1000th-tile where noncarriers' mean phenotype with 95% confidence interval, exceeds carriers' mean phenotype with 95% confidence interval.

File Name: Supplemental Data 5.
Description: Enrichment of noncarriers with extreme PRSs within individuals that meet disease thresholds. Work from Ripatti, et al. was replicated within the UKB 200K exomes and shows that within individuals that meet disease thresholds, there is an enrichment of noncarriers with PRS in the 90th percentile and higher. * indicates that PRS extreme was defined as the bottom 10th percentiles.

File Name: Supplemental Data 6.
Description: Detecting marginal epistasis using PRS*carrier status interaction terms. Phenotypes were regressed against 1st 10 genetic PCs, sex, age, PRS, carrier status, and PRS*carrier status to identify potential marginal epistasis modifying carrier phenotype, but did not discover additional interactions from those previously published. p-values generated from linear regression; * indicates significance after Bonferroni corrections (p<0.05/5=0.01).

File Name: Supplemental Data 7.
Description: Full marginal epistasis with monogenic genes results. σ^2_G, variance in phenotype explained by genetics and σ^2_G SE, its standard error. β^2_C, variance in phenotype due to carrier status, and β^2_C SE its standard error. σ^2_{CxG}, variance in phenotype explained by marginal epistasis between genetic background and carrier status and σ^2_{CxG} SE its standard error. σ^2_e, the variance in phenotype. Chip heritability, the ratio of σ^2_G and σ^2_e. EIP ratio, σ^2_{CxG}/β^2_C, and its standard error, EIP ratio SE. *p*-values generated from FAME method, * indicates significance after Bonferroni corrections (less than p=0.05/6=0.0083).
