# Supplementary material for: Investigating the sources of variable impact of pathogenic variants in monogenic metabolic conditions
Source: Nat Commun. 2025 Jun 5;16:5223. doi: 10.1038/s41467-025-60339-7 (PMC12141715; doi:10.1038/s41467-025-60339-7)
Supplement: Supplementary file 4 — Reporting Summary [file 41467_2025_60339_MOESM4_ESM.pdf]

nature portfolio

Corresponding author(s): Valerie Arboleda, Noah Zaitlen

Last updated by author(s): Apr 10, 2025

Reporting Summary

Nature Portfolio wishes to improve the reproducibility of the work that we publish. This form provides structure for consistency and transparency in reporting. For further information on Nature Portfolio policies, see our Editorial Policies and the Editorial Policy Checklist.

Statistics

For all statistical analyses, confirm that the following items are present in the figure legend, table legend, main text, or Methods section.

n/a	Confirmed	
☐	☒	The exact sample size (*n*) for each experimental group/condition, given as a discrete number and unit of measurement
☐	☒	A statement on whether measurements were taken from distinct samples or whether the same sample was measured repeatedly
☐	☒	The statistical test(s) used AND whether they are one- or two-sided<br>*Only common tests should be described solely by name; describe more complex techniques in the Methods section.*
☐	☒	A description of all covariates tested
☐	☒	A description of any assumptions or corrections, such as tests of normality and adjustment for multiple comparisons
☐	☒	A full description of the statistical parameters including central tendency (e.g. means) or other basic estimates (e.g. regression coefficient) AND variation (e.g. standard deviation) or associated estimates of uncertainty (e.g. confidence intervals)
☐	☒	For null hypothesis testing, the test statistic (e.g. *F*, *t*, *r*) with confidence intervals, effect sizes, degrees of freedom and *P* value noted<br>*Give P values as exact values whenever suitable.*
☒	☐	For Bayesian analysis, information on the choice of priors and Markov chain Monte Carlo settings
☒	☐	For hierarchical and complex designs, identification of the appropriate level for tests and full reporting of outcomes
☐	☒	Estimates of effect sizes (e.g. Cohen's *d*, Pearson's *r*), indicating how they were calculated

Our web collection on statistics for biologists contains articles on many of the points above.

Software and code

Policy information about availability of computer code

Data collection	No software was used in data collection.
Data analysis	Software used is cited in Methods section and all are open source: Plink v1.9 & 2.0, R v4.1.1, Ensembl Variant Effect Predictor v107, FAME v1.0. Code will be available in GitHub in https://github.com/angela-wei/penetrance_expressivity.

For manuscripts utilizing custom algorithms or software that are central to the research but not yet described in published literature, software must be made available to editors and reviewers. We strongly encourage code deposition in a community repository (e.g. GitHub). See the Nature Portfolio guidelines for submitting code & software for further information.

Data

Policy information about availability of data

All manuscripts must include a data availability statement. This statement should provide the following information, where applicable:

- Accession codes, unique identifiers, or web links for publicly available datasets
- A description of any restrictions on data availability
- For clinical datasets or third party data, please ensure that the statement adheres to our policy

UK Biobank access was obtained via https://www.ukbiobank.ac.uk/enable-your-research. BioMe access was obtained via requests submitted to BioMe Biobank and Mount Sinai Data Warehouse.

April 2023

Databases also used in this work include: ClinVar (https://www.ncbi.nlm.nih.gov/clinvar/), gnomAD exomes v2.1 (https://gnomad.broadinstitute.org/), Cardiovascular Disease KP genetic association datasets (https://cvd.hugeamp.org/datasets.html), Global Lipids Genetics Consortium Results (https://csg.sph.umich.edu/willer/public/glgc-lipids2021/), PubMed (https://pubmed.ncbi.nlm.nih.gov/), and GoogleScholar (https://scholar.google.com/).

Research involving human participants, their data, or biological material

Policy information about studies with human participants or human data. See also policy information about sex, gender (identity/presentation), and sexual orientation and race, ethnicity and racism.

Reporting on sex and gender	Biological sex was included as a covariate in analyses. Sex distribution reported in Table 1.
Reporting on race, ethnicity, or other socially relevant groupings	Missense variant analysis was inclusive of all individuals in UK Biobank and BioMe with exomes available. Polygenic risk score and marginal epistasis analysis was restricted to unrelated individuals of European ancestry.
Population characteristics	UK Biobank is made up of data collected from about 500,000 individuals aged from 40-69 from the United Kingdom. Genotype, phenotype, and health record data of these individuals were collected over the course of 2006-2010. Our analysis was focused on 200,632 participants with exome sequences available and cardiometabolic traits (LDL, HDL, triglycerides, HbA1C, body mass index) measured. These individuals were 55.1% female and had a mean age of 56.5 years at recruitment (Table 1).<br><br>BioMe Biobank is made up of over 50,000 individuals within the Mount Sinai health system. We focused on 28,817 with exome sequencing available. Of these participants, 59.3% were female and the median age at recruitment was 59 years.
Recruitment	UK Biobank recruited volunteers for population-level study - details here https://www.ukbiobank.ac.uk/enable-your-research/about-our-data/baseline-assessment.<br>UK Biobank recruited volunteers as detailed here https://www.ukbiobank.ac.uk/enable-your-research/about-our-data/baseline-assessment.<br><br>BioMe Biobank recuited individuals from clinical care sites.
Ethics oversight	*Identify the organization(s) that approved the study protocol.*

Note that full information on the approval of the study protocol must also be provided in the manuscript.

Field-specific reporting

Please select the one below that is the best fit for your research. If you are not sure, read the appropriate sections before making your selection.

☒ Life sciences ☐ Behavioural & social sciences ☐ Ecological, evolutionary & environmental sciences

For a reference copy of the document with all sections, see nature.com/documents/nr-reporting-summary-flat.pdf

Life sciences study design

All studies must disclose on these points even when the disclosure is negative.

Sample size	No sample size was pre-determined, sample sizes were based on individuals with both exome and phenotype data available.<br>200,628 individuals were included from UK Biobank and 28,817 individuals were included from UK Biobank.
Data exclusions	When completing polygenic risk scores and marginal epistasis analyses, we restricted to European, unrelated individuals with genotyping, exome, and phenotyping data.
Replication	Missense variant analysis was replicated in the BioMe biobank (Figure 3b).
Randomization	No randomization was included in this study. Individuals were divided into groups based on carrier status of pathogenic variants. Covariates were included into our analyses (see Methods), including age, sex, and first 10 genetic principal components.
Blinding	No blinding occurred in this study.

Reporting for specific materials, systems and methods

We require information from authors about some types of materials, experimental systems and methods used in many studies. Here, indicate whether each material, system or method listed is relevant to your study. If you are not sure if a list item applies to your research, read the appropriate section before selecting a response.

Materials & experimental systems

n/a	Involved in the study
☒	☐ Antibodies
☒	☐ Eukaryotic cell lines
☒	☐ Palaeontology and archaeology
☒	☐ Animals and other organisms
☒	☐ Clinical data
☒	☐ Dual use research of concern
☒	☐ Plants

Methods

n/a	Involved in the study
☒	☐ ChIP-seq
☒	☐ Flow cytometry
☒	☐ MRI-based neuroimaging

Plants

Seed stocks	*Report on the source of all seed stocks or other plant material used. If applicable, state the seed stock centre and catalogue number. If plant specimens were collected from the field, describe the collection location, date and sampling procedures.*
Novel plant genotypes	*Describe the methods by which all novel plant genotypes were produced. This includes those generated by transgenic approaches, gene editing, chemical/radiation-based mutagenesis and hybridization. For transgenic lines, describe the transformation method, the number of independent lines analyzed and the generation upon which experiments were performed. For gene-edited lines, describe the editor used, the endogenous sequence targeted for editing, the targeting guide RNA sequence (if applicable) and how the editor was applied.*
Authentication	*Describe any authentication procedures for each seed stock used or novel genotype generated. Describe any experiments used to assess the effect of a mutation and, where applicable, how potential secondary effects (e.g. second site T-DNA insertions, mosiacism, off-target gene editing) were examined.*
